# Supplementary material for: Engineering bioactive nanoparticles to rejuvenate vascular progenitor cells
Source: Commun Biol. 2022 Jun 29;5:635. doi: 10.1038/s42003-022-03578-4 (PMC9243106; doi:10.1038/s42003-022-03578-4)
Supplement: Supplementary file 2 — Supplementary Information [file 42003_2022_3578_MOESM2_ESM.pdf]

Supplementary Materials for

Engineering Bioactive Nanoparticles to Rejuvenate Vascular Progenitor Cells

Loan Bui[1], Shanique Edwards[2], Eva Hall[1], Laura Alderfer[1], Kellen Round[1], Madeline Owen[1], Pietro Sainaghi[1], Siyuan Zhang[3,4], Prakash D. Nallathamby[1], Laura S. Haneline[2,5,6], Donny Hanjaya-Putra[1,4,7,8]

*Corresponding author. Email: dputra1@nd.edu

This PDF file includes:

Supplementary Methods
Supplementary Figures S1 to S12
Supplementary Tables S1 to S3

Other Supplementary Materials for this manuscript include the following:

Movies S1 to S8

Supplementary Methods

Nanoparticle Fabrication

Synthesis of multilamellar liposomal nanoparticles (NPs) was performed based on the standard thin film lipid hydration method.[33,34] The components consist of phospholipids (Avanti® Polar Lipids) including MPB-PE (1,2-dioleoyl-sn-glycero-3-phosphoethanolamine-N-[4-(p-maleimidophenyl)butyramide] (sodium salt)), DOPC (1,2-dioleoyl-sn-glycero-3-phosphocholine), DOPG (1,2-dioleoyl-sn-glycero-3-phospho-(1'-rac-glycerol) (sodium salt), fluorescent tracker 1,1′-Dioctadecyl-3,3,3′,3′-tetramethylindocarbocyanine perchlorate (Dil, Sigma-Aldrich), and SB-431542 (SB, Stemcell Technology) (**Supplementary Fig. 1A**). DOPC, DOPG, MBP-PE, SB-43154 and 'Dil' dye lipid components from Avanti Lipids Inc. were combined in a glass vial and vacuum dried (-25 mm of Hg, 21°C) for 30 to 45 minutes to obtain the dry lipid film. To fabricate liposomal nanoparticles, the dry lipid film containing DOPC/DOPG/MPB-PE/Dil/SB (1188/303/1890/120/3.6µg) was hydrated with 1ml PBS solution. The resulting mixture was vortexed for 5-7 min, then extruded 21 times using gas tight syringes, through a 200nm polycarbonate membrane sandwiched in the Mini-Extruder block (Avanti® Polar Lipids) to improve monodispersity of small liposomal vesicles (<200 nm). The resulting solution was incubated at room temperature for 1.5 hr, then at 4°C overnight. The NPs were purified from free phospholipids and free SB by ultracentrifugation at 50,100 rpm at 4ºC for 2.5hr (ThermoScientific, Sorvall MX120+ Micro-Ultracentrifuge, Rotor S55-A2). The SB-NPs were obtained by resuspending the pellet in PBS.

Nanoparticle Characterization

Near-UV absorbance of SB-431542 was examined to determine the maximal absorbance wavelength for SB-431542. A solution of SB-431542 in PBS (20µM) was prepared and added to a UV-Star® 96-well plate (Greiner Bio-One, 675801) before measurement. The absorbance profile (at 250nm to 400nm) was measured using a plate reader (Synergy H1, BioTek). To generate a standard curve, SB was diluted in PBS solution to have concentrations of 0, 0.3125, 0.625, 1.25, 2.5, 5, 10, and 20 µM. Absorbances were measured at 320 nm and results were plotted. The resulting standard curve was used to calculate the concentrations of SB in the release study. Based on the near-UV absorbance profile, maximal absorbance of SB-431542 in PBS was observed at 320-324nm (**Supplementary Fig. 4A**). Using this maximum absorbance, a linear standard curve was generated (**Supplementary Fig. 4B).** This curve was utilized to calculate the encapsulation efficiency and amount of SB released by measuring the SB-431542 amount in the supernatant after centrifugation. To determine the encapsulation efficiency, the supernatants (n=3) were preserved after the ultracentrifugation step described above. The amount of non-encapsulated SB was determined by measuring the absorbance at 320nm (Synergy H1, Biotek) (**Supplementary Fig. 4**). The encapsulation efficiency of the nanoparticles was calculated using the following equation.

$$\text{Encapsulation Efficiency} = \frac{(\text{Total amount of SB used} - \text{Non encapsulated SB})}{\text{Total amount of SB used}} \text{x } 100\%$$

The NPs hydrodynamic radius was quantified by dynamic light scattering (DLS, Zen 3600, Malvern) and nanoparticle tracking analysis (Nanosight, NS3000, Malvern). The NPs morphology was further validated by cryo-transmission electron microscopy (cryo-TEM, Jeol 2011). The stability of SB-NPs at both 37°C and 4°C was assessed by DLS measurement over 30 days. For

the study of SB release, the suspension of SB-NPs in PBS was incubated at 37°C. Each day, the suspension was ultra-centrifuged at 50,000 rpm at 4°C for 2.5 hr. The supernatant was then collected, and the pellet was resuspended in PBS. Released SB was quantified from the supernatant via an absorbance measurement at 320nm.

Cell Surface Conjugation and Characterization

In order to engineer the cells with NPs via maleimide-thiol coupling, the presence of thiol groups on cell surfaces was validated. The cells were stained with Oregon Green TM 488 Maleimide. Stained cells were observed using fluorescent microscopy and acquired on a flow cytometer. Expression of thiol groups on the cell surface of the two cell types (ECFC and GDM-ECFC) was comparable, as well as the mean fluorescent intensity (MFI) of the stained cells (**Supplementary Fig. 3**). To identify and quantify the presence of thiol groups (-SH) on the ECFC cell surface, the cells were stained with Oregon Green 488 Maleimide and analyzed using flow cytometry. Suspended cells (10^6 cells) were washed twice with 1% BSA in PBS, then stained with Oregon Green 488 Maleimide (4 µg/ml) for 15 min at room temperature. The cells were again washed twice with 1% BSA in PBS. Afterwards, the cells were fixed with 1% paraformaldehyde and imaged with a fluorescent microscope (Revolve, Echo). Mean fluorescence intensity of the cells was quantified using flow cytometry (BD LSR Fortessa X-20, FITC channel).

Drug-loaded NPs were conjugated on the surface of the cells by mixing equal volumes of ECFCs (3 x 10^6 cells/mL in EGM-2 basal medium) and SB-NPs in nuclease-free water, with nanoparticles to cell ratios ranging from 100 to 5,000. The cells were then incubated for 30 min at 37°C with gentle agitation every 10 min to facilitate the conjugation of maleimide on MPB-PE in liposomes to the free thiols on ECFCs. After a PBS wash to separate cells from the unbound nanoparticles, the residual maleimide groups on cell-bound particles were quenched by

incubation of 3 x 10^6 cells/mL with 1 mg/mL thiol-terminated 2-kDa PEG (Laysan Bio) at 37C for 30 mins in complete EGM-2 medium, followed by two PBS washes to remove unbound PEG. After nanoparticles were conjugated on the cell surface, their presence was confirmed by confocal microscopy (Nikon A1R-MP). Mean fluorescence intensity (MFI) was measured using Flow Cytometry (BD LSR Fortessa X-20) and FlowJo software.

Isolation and Characterization of ECFCs

Human umbilical cord blood samples (40-60 mL) were collected in heparinized solution at the time of birth for normal / uncomplicated and GDM pregnancies (gestational age 38-42 weeks) following written informed consent.[3,4,19] GDM was defined per American College of Obstetrics and Gynecology guidelines. Exclusion criteria include T1DM or T2DM, illness known to affect glucose metabolism (i.e., Cushing syndrome, polycystic ovarian syndrome), use of medications that affect glucose metabolism (i.e., dexamethasone), multiple gestation, history of pre-eclampsia, cardiovascular disease, and women carrying fetuses with chromosomal abnormalities. Historical and clinical data were obtained at each visit and at the time of delivery (**Supplementary Table 1**). Maternal blood was collected for glycosylated hemoglobin (HgA1C) and oral glucose tolerance test (GTT). Cord blood was collected and processed in our AngioBioCore facility for human mononuclear cells (MNCs) used for ECFC isolation and assays. The Institutional Review Board at the Indiana University School of Medicine (IUSM) approved all protocols, and informed consent was obtained from all women.

The human ECFCs were isolated and characterized as previously described.[3,9,19,67] Briefly, tissue culture plates pre-coated with collagen I were seeded with human MNCs in complete endothelial growth medium-2 (EGM-2). After 24 hours of culture, nonadherent cells were aspirated and complete EGM-2 medium was added to each well. Colonies of endothelial cells

appeared between 5 and 8 days and were identified as monolayers of cobblestone-appearing cells. ECFCs were characterized for the positive expression of cell-surface antigens CD31, CD141, CD105, Cd144, vWF, and Flk-1, as well as negative expression of hematopoietic-cell surface antigens CD41 and CD14. Single cell colony forming assays were used to characterize their robust and proliferative potential, secondary and tertiary colony formation upon plating. Normal or uncomplicated ECFC lines include E1-CB-111, E1-CB-150, E1-CB-153, E1-CB-157; GDM-ECFC lines include E1-CB-36, E1-CB-37, E1-CB-71, and E1-CB-74 (**Supplementary Table. 1)**. To maintain the ECFC culture, flasks or well plates were pre-coated with rat-tail collagen type I solution (50µg/mL, Corning), then incubated for at least 3 h at 37°C and washed with PBS three times before use. Complete medium for growing ECFCs consisted of EGM-2 (Promocell, C-22011), supplement Mix (Promocell, C-39216), and 0.2% mycoZapTM Prophylactic (Lonza). The cells were maintained at 37°C, 5% CO_2, passaged using DetachKit (Promocell, C-41222), and used for experiments between passages 2-5. All cell lines were routinely tested for mycoplasma contamination and were negative throughout this study.

Cell Growth and Proliferation Assays

Cell growth over time was determined by Alamar Blue™ Cell Viability Reagent (Thermo Fisher Scientific) following the manufacturer's protocol. A standard curve was created by seeding cells on 96-well plates with different predetermined cell numbers. Cells were incubated for 24 hr then culture medium was replaced by Alamar Blue containing medium. After 1-2 hours of incubation, the absorbances of different cell-containing wells were measured at 540nm with the reference wavelength at 600nm using the plate reader (The VICTOR3 Multilabel Plate Reader, PerkinElmer). Standard curves were generated and used to calculate cell numbers based on absorbance measurements. To determine cell growth of NP-conjugated cells, ECFC cells

conjugated with different NP ratios were seeded with an initial density of 10,000 cells/well. Unconjugated cells served as the control. The number of cells were determined at day 1, 2, 3 and each condition was measured in triplicates.

Cell Viability Assay

Cells were seeded at a concentration of 5,000 cell/well in a 96 well plate. Cells were incubated for 48 hrs. The cells were stained with Calcein AM and Ethidium Homodimer-1 (Thermo Fisher Scientific) as determined by the manufacturer’s protocol. The ratio of Calcien AM (green) to total number of cells (dead cells in red and green) was used to determine the percentage of live cells. This was normalized to the untreated group.

Flow cytometry

In order to examine the expression of endothelial cell surface markers, flow cytometry was performed following standard procedure.[9] Briefly, suspended cells (10^6 cells) were washed twice with 1% BSA in PBS, then stained with the antibodies (1µg/ml) for 30 min at room temperature: Anti-CD31 antibody [B-B38] FITC (Abcam, ab27333), Anti-CD34 antibody [ICO-115] (Phycoerythrin) (Abcam, ab187284), and Anti-CD144 antibody [EPR1792Y] Alexa Fluor® 488 (Abcam, ab195202), as well as with their corresponding IgG isotype controls (**Supplementary Table. 3**). The cells were again washed twice with 1% BSA in PBS. Then, the biomarkers’ expression was determined using flow cytometry (BD LSR Fortessa X-20) and the MFI was analyzed using FlowJo.

In order to examine whether the NPs were retained on the surface of the cells over numerous cell divisions, cells were first labeled with CFSE (Abcam, ab113853). NPs-conjugated

cells were then obtained by conjugating the CFSE-labeled cells with NPs containing the Dil fluorescent tracker whereas control cells did not have NPs. Both NPs-conjugated cells and control cells were collected daily for 4 consecutive days. Flow cytometry (BD LSR Fortessa X-20) was performed to determine the CFSE (FITC channel) and Dil (PE-A channel) signals. The MFI of both CFSE and Dil signals were analyzed using FlowJo.

NPs were conjugated to the surface of cells at a ratio of 1 cell to 5,000 NPs. These cells were seeded into a T75 flask. At the same time, untreated cells of the same cell line and passage number were seeded into another T75. These cells were allowed to grow for 24 hours at 37°C, 5% CO_2. After that, the supernatant was removed from the NP conjugated cells, filter through a 0.2 μM filter, and added to the untreated cells. The NP conjugated cells received fresh media. The cells were incubated for another 24 hours at 37°C, 5% CO_2. Flow cytometry (BD LSR Fortessa X-20) was performed to determine the Dil (APC channel) signal.

Gene Expression

All normal ECFCs and GDM-ECFCs at passage 3-5 were used for studying gene expression. Cells were plated on the 6-well plates precoated with collagen type I as described above. Treated cells were subjected to culture medium with 5 μM SB (treated medium), whereas vehicle control cells were incubated with a medium containing the same amount of DMSO (vehicle control). After 72 hr of incubation, as cells reached 70-80% confluency, RNA was collected for further analysis of *TAGLN* expression. To mimic the transient effect of SB in the body, an experiment was set up in a similar way with ECFCs from normal and GDM pregnancies. After the first 72 hr of incubation (**Fig 3B**), at day 4 all media was replaced by fresh media (i.e. SB was removed from the treated medium) and incubation was continued for 3 more days. RNA was then

collected at days 4, 5, and 6 for further analysis (**Fig 3B**) of *TAGLN* expression. To study the gene expression of cells treated with SB-NPs, ECFCs and GDM-ECFCs at passage 3-5 were conjugated with either vehicle (DMSO) NPs (Vh-NPs) or SB-NPs. Cells were plated on the 6-well plates or 10mm petri dish precoated with collagen type I, incubated for 6 days. As cells reached 70-80% confluency, RNA was collected for further analysis of *TAGLN* expression.

RNA was isolated and purified using TRIzol® Reagent (Life technologies) and RNeasy® Plus Mini Kit (Qiagen), respectively.[40] RNA concentrations and purity were determined using the NanoDropTM 2000c Spectrophotometer (Thermo Fisher Scientific) with RNase-free water as the blank. Reverse transcription was performed using the High-Capacity cDNA Reverse Transcription Kit (Applied Biosystems) according to the manufacturer's protocol. Quantitative real-time reverse transcription PCR (qRT-PCR) was performed using TaqMan primer for *TAGLN* (assay ID Hs01038777_g1) and the endogenous control gene *HPRT-1* (assay ID Hs02800695_m1). The 384-well plate was sealed and loaded into an qRT-PCR machine (QuantStudioTM 5 System, Applied Biosystems) and run by a fast mode cycle.

Western Blot

Whole cell lysates were collected using RIPA buffer containing protease inhibitor cocktails 2 and 3 (Sigma Aldrich). 10 µg of protein per sample was loaded into a 10% Bis-Tris pre-cast gel (Life Technologies). Proteins were transferred to a nitrocellulose membrane and immunoblotted for transgelin (1:5000, ab14106, Abcam), and vinculin (1:10,000, VIN-11-5, Sigma Aldrich), followed by horseradish peroxidase (HRP)-conjugated secondary antibodies (goat anti-rabbit and goat anti-mouse, respectively). Blots were developed using SuperSignal™ West Femto Maximum Sensitivity Substrate (Thermo Fisher), and quantified using ImageJ.

Migration Assay

Cell migration was examined using the transwell assay (**Supplementary Fig. 7**).[12] Transwell inserts (Falcon™ Cell Culture Inserts 08-771-21, pore size 8μm) were placed into a 24-well plate and pre-coated with collagen I. Collagen-coated transwells were washed with PBS and allowed to air-dry in the biosafety hood. 30,000 cells per well were seeded in the top portion of the transwell insert. Migration was stimulated by a 10% FBS gradient added to the bottom of the wells. The transwell plate was incubated at 37°C, 5% CO_2. After a 4hr incubation period, the top part of each transwell was wiped with a cotton swab to remove non-migrated cells. Migrated cells at the bottom of the transwells were fixed with 4% paraformaldehyde for 15 min at 37°C, then washed with PBS. Fixed cells were stained with 1% Crystal Violet in 10% Acetic Acid at room temperature for 10-15 min, then washed with PBS. Seven to eight random areas were imaged per condition and quantified to determine the number of migrated cells in the assay.

Wound Healing Assay

The 2-well culture inserts (ibidi) were placed in each well of a 24-well plate precoated with rat-tail collagen Type 1 solution (50μg/mL, Corning). Cells were seeded inside each chamber and incubated for two days to reach confluence. At this point, the culture inserts were removed to create scratch areas and imaging was initiated (Lionheart FX Automated Microscope, BioTek) to visualize the wound closure process. Wound confluency was measured in 30 min increments for 14 hr. Data was obtained from Gen5 software (BioTek); the average from two cell lines of each group, normal ECFCs and GDM-ECFCs was analyzed using GraphPad.

Matrigel Assay

To visualize network formation *in vitro*, a Matrigel vasculogenesis assay was performed using a 15-well angiogenesis plate (µ-Slide Angiogenesis, ibidi).[51] Each well was coated with Matrigel and incubated at 37°C for at least 2 hr. Cells were then seeded onto each Matrigel-containing well at a density of 4,000 cells per well. Network formation was visualized and imaged every 30 minutes for 10 hr (Lionheart FX Automated Microscope, BioTek). Analysis was performed using the KAV plugin in the FIJI program.[31,80] Data was analyzed using GraphPad.

Collagen/Fibronectin Gels for in vitro study

Cells (10^6 per ml) conjugated with either Vh-NPs or SB-NPs were suspended in a solution of rat-tail collagen Type 1 solution (1.5mg/ml, Corning) and human plasma fibronectin (100ng/ml, Sigma-Aldrich) at pH 7.4. The cell-gel suspension was pipetted into 48-well plates (250 µl/well, i.e. 250,000 cells per well) and incubated at 37°C for 30 mins to allow polymerization. Then 500µl EGM medium was added to each well. To visualize the branching network and lumen formation, bright field images of the cell-containing gel plug were taken before implantation. Extra gel plugs were fixed with 4% paraformaldehyde and subsequently stained with Phalloidin, Rhodamine-conjugated *Ulex Europaeus Agglutinin* I (UEA-1), and DAPI (**Supplementary Fig. 9**) and fluorescent images were acquired at 20X (Revolve, Echo microscope). Analysis was performed using the KAV plugin in the FIJI program.[31,40,80] Data was analyzed using GraphPad Prism.

Collagen/Fibronectin Gels for in vivo study

Subcutaneous implantation of the cell-containing gel plugs was conducted with 8-week old NOD-SCID mice following the procedure approved by Indiana University School of Medicine

IACUC. Briefly, mice were anesthetized by isoflurane. A small incision was made to create two subcutaneous pockets near the dorsal flanks. One gel plug was inserted into each pocket, one with Vh-NPs and one with SB-NPs. The incision was clipped and Ketoprofen (100mg/ml) was injected for reducing pain. On day 14, the mice were euthanized and the gel plugs were harvested and fixed with 4% paraformaldehyde. Tissue samples were processed, sectioned and stained for H&E at the Histology and Histomorphometry Core, Indiana Center for Musculoskeletal Health, IUSM. Sectioned slides were then stained for H&E and IHC using human CD31 (clone JC70A, Dako), mouse CD31, and mouse SMA, as well as appropriate IgG isotype controls (**Supplementary Fig. 11**). The number of human blood vessels and size were counted, measured, and normalized to the graft area. We sampled a minimum of 10 images for each graft, analyzed, and normalized the number and size of blood vessels accordingly.

Vessel Perfusion Study and Intravital Microscopy

Subcutaneous implantation of the cell-containing gel plugs was conducted with 8-week NOD-SCID mice following the procedure that was approved by Notre Dame Research Compliance. Briefly, mice were anesthetized by isoflurane. A small incision was made to create two subcutaneous pockets near the dorsal flanks. One gel construct was implanted into each pocket; one side with Vh-NPs and the other side with SB-NPs. The incision was clipped and Ketoprofen (100mg/ml) was injected to reduce pain. A perfusion study was performed on day 14. Rhodamine-conjugated *UEA-1* (Vector Laboratory, dilution ratio 1:2) and FITC-conjugated *GS-IB4* solutions (Vector Laboratory, dilution ratio 1:2) were injected through retro-orbital veins.[41] After 20 mins, intravital images were taken using a dissecting microscope (Leica, magnification 12X) and multiphoton microscope (Olympus). Confocal image stacks were acquired to create 3D rendering images, which were quantified for percent area covered by human and mouse vessels,

as well as for distribution of vessel diameters using ImageJ. At the end of the experiment, the grafts were harvested, fixed with 4% paraformaldehyde, and processed for H&E staining and IHC analysis (**Supplementary Fig. 12**). Images for IHC analysis were acquired with a brightfield microscope (Revolve, Echo microscope, 10X magnification).

Statistical Analysis

All experiments were performed in at least three independent experiments, conducted in triplicate with at least 4 biological replicates. Data are presented as mean ± standard deviation, unless otherwise were specified in the figure legends. A power analysis with a 95% confidence interval was used to calculate sample size required to obtain statistically significant results. The sampling number we used gave a normal distribution. All statistical analysis were conducted in GraphPad Prism. Statistical comparisons were made using Student's *t* test for paired data, analysis of variance (ANOVA) for multiple comparisons, and with Tukey post hoc analysis for parametric data. Significance levels were set at the following: *$P<0.05$, **$P<0.01$, ***$P<0.001$, ****$P<0.0001$.

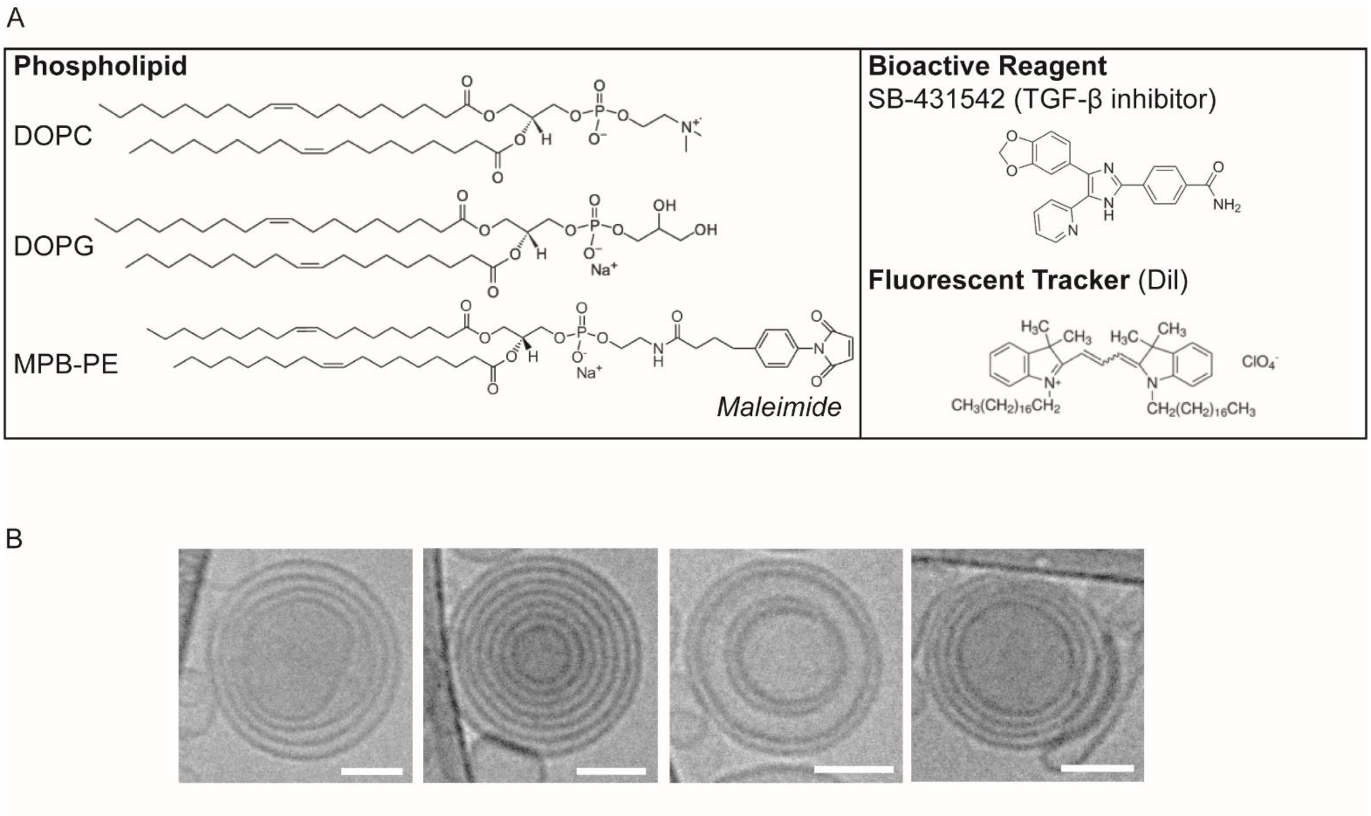

Supplementary Figure 1. (**A**) Chemical components of the bioactive nanoparticles. (**B**) Cryo-TEM images of synthesized multilamellar nanoparticles. Scale bars are 50 nm.

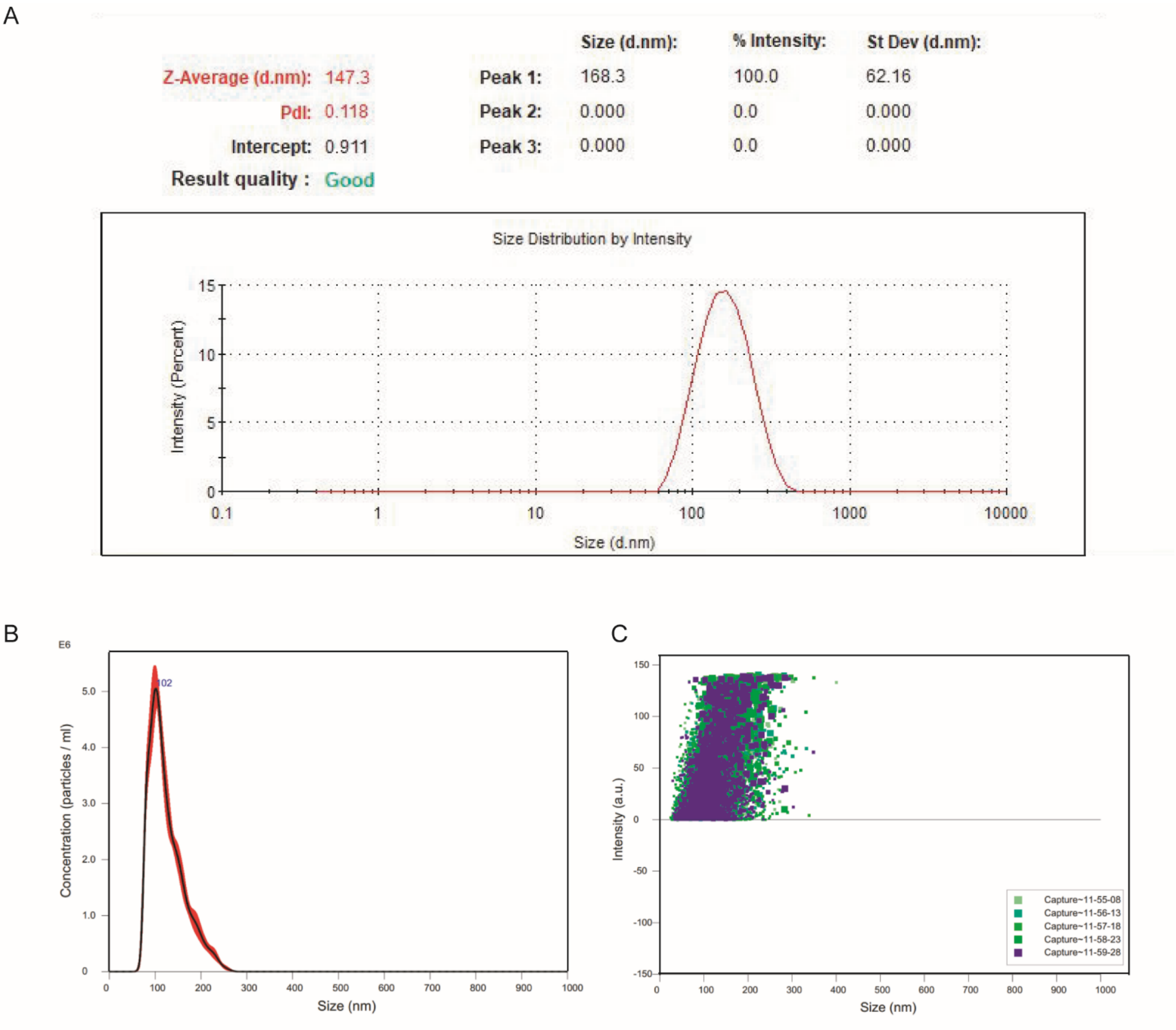

Supplementary Figure 2. **Nanoparticle Characterization.** (**A**) Representative data of DLS for particle's hydrodynamic diameter and their monodispersity. (**B**) Representative data of Nanosight for confirming the particle's size and (**C**) providing measurement of NPs concentration.

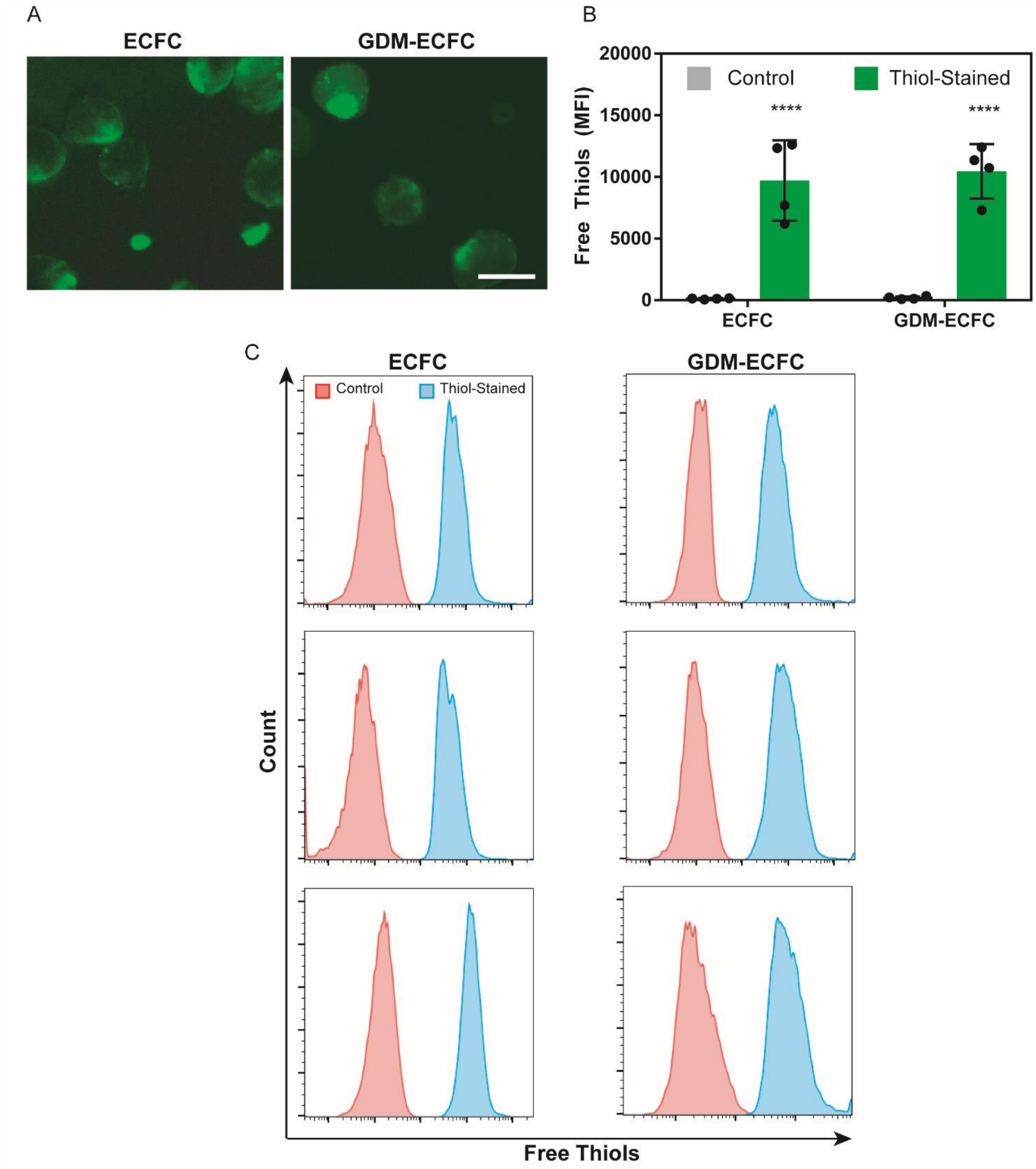

Supplementary Figure 3. **Cell surface free thiols.** (**A**) Representative fluorescent images of ECFC and GDM-ECFC stained with Oregon Green^{TM} 488 Maleimide. Scale bar: 10µm. (**B**) Quantification of Thiol expression, mean fluorescence intensity (MFI) showed no significant difference between ECFC and GDM-ECFC. **** P<0.001 for comparison of Thiol stained and control unstained cells. (**C**) Histogram data for cell surface thiols of individual cell line comparing the control cells (red) and stained cells (blue)

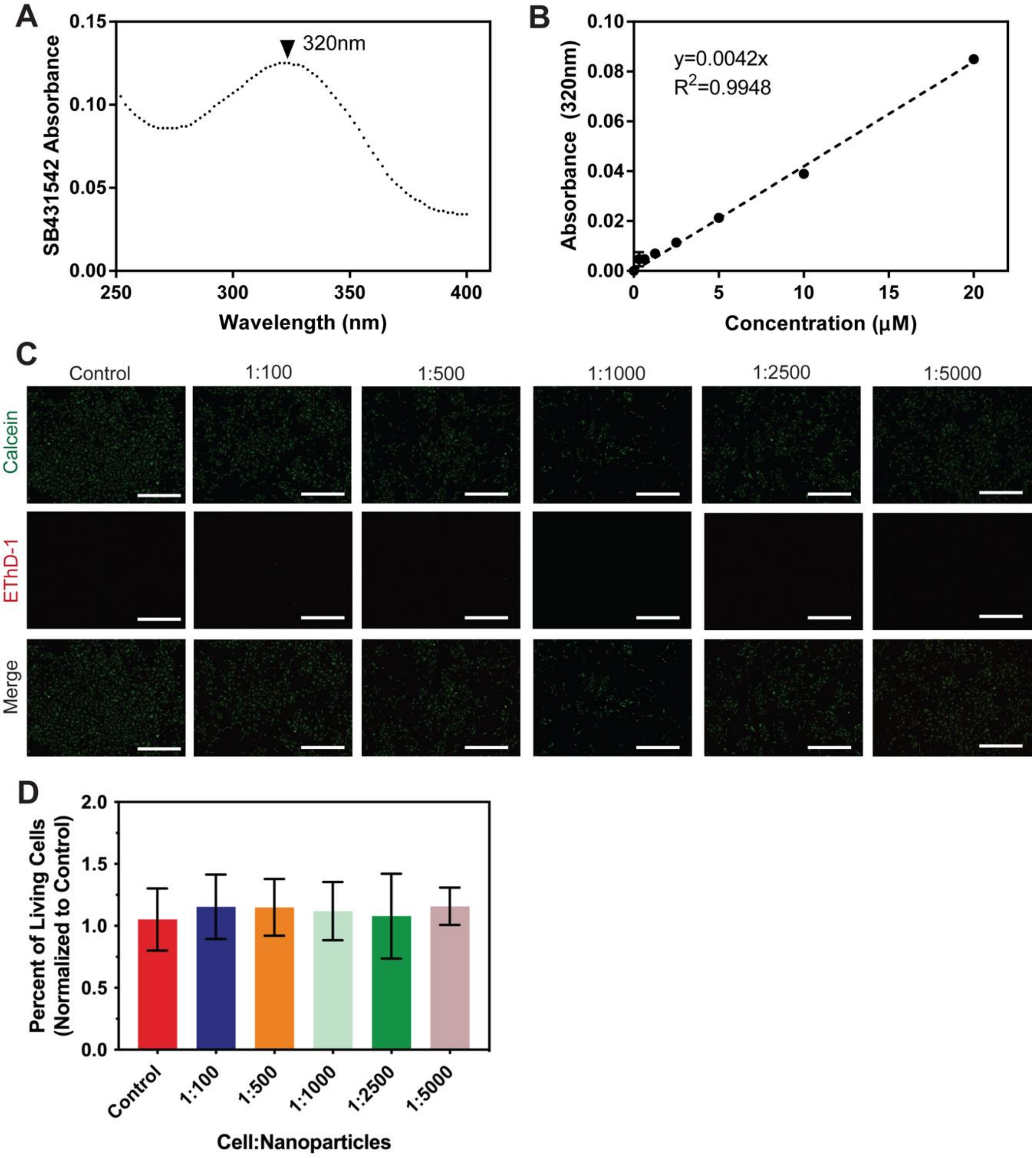

Supplementary Figure 4. (**A**) Near-UV absorptivity profile of SB-431542 in PBS solution. Maximal absorbance was observed at 320 nm. (**B**) Standard curve of SB-431542 absorbance at different concentrations. (**C**) SB-431542 containing NPs were conjugated onto the surface of ECFCs with various cell to NP ratios (1:100, 1:500, 1:1000, 1:2500, and 1:5000). After 48 hours of culture, ECFCs were stained with Calcein AM (green) and Ethidium homodimer-1 (red). Representative images indicate cell viability of unconjugated ECFCs control and ECFCs with various cell to NP ratios. Scale bars are 1mm. (**D**) Image J was used to analyze the fluorescent images. Compared to unconjugated ECFCs control, no significant differences in cell viability was observed among groups with varying cell to NP ratios. n=3; mean ± s.d. of two independent experiments conducted in triplicate.

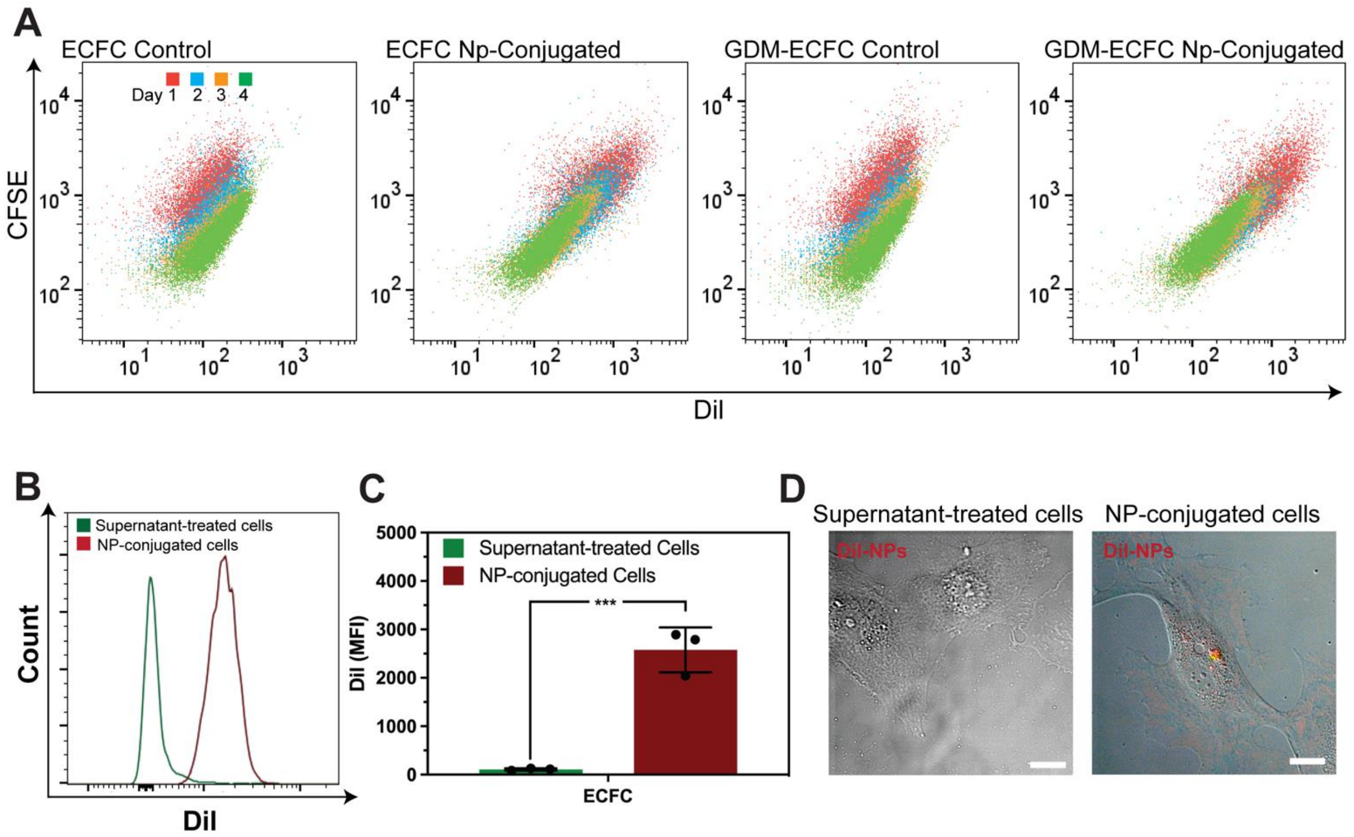

Supplementary Figure 5. **(A)** Dot-plot of CFSE and Dil (Np) signals for both normal ECFC and GDM-ECFC. Control cells are non-conjugated. **(B)** Representative histogram flow cytometry data for supernatant-treated ECFCs (green) and NP-conjugated ECFCs (red). Multilamellar lipid nanoparticles were labeled with Dil-dyes. **(C)** Quantification of Dil-labeled nanoparticles conjugated onto ECFCs. Mean fluorescence intensity (MFI) showed no significant expression of Dil-NPs on the supernatant-treated cells. *n*=3; mean ± s.d. of two independent experiments conducted in triplicate. Statistical significance was set at $^{***}P<0.005$. **(D)** The Representative fluorescent images of supernatant-treated ECFCs and NP-conjugated ECFCs. Multilamellar lipid nanoparticles (NPs) were labeled with Dil-dyes. Scale bars are 10µm.

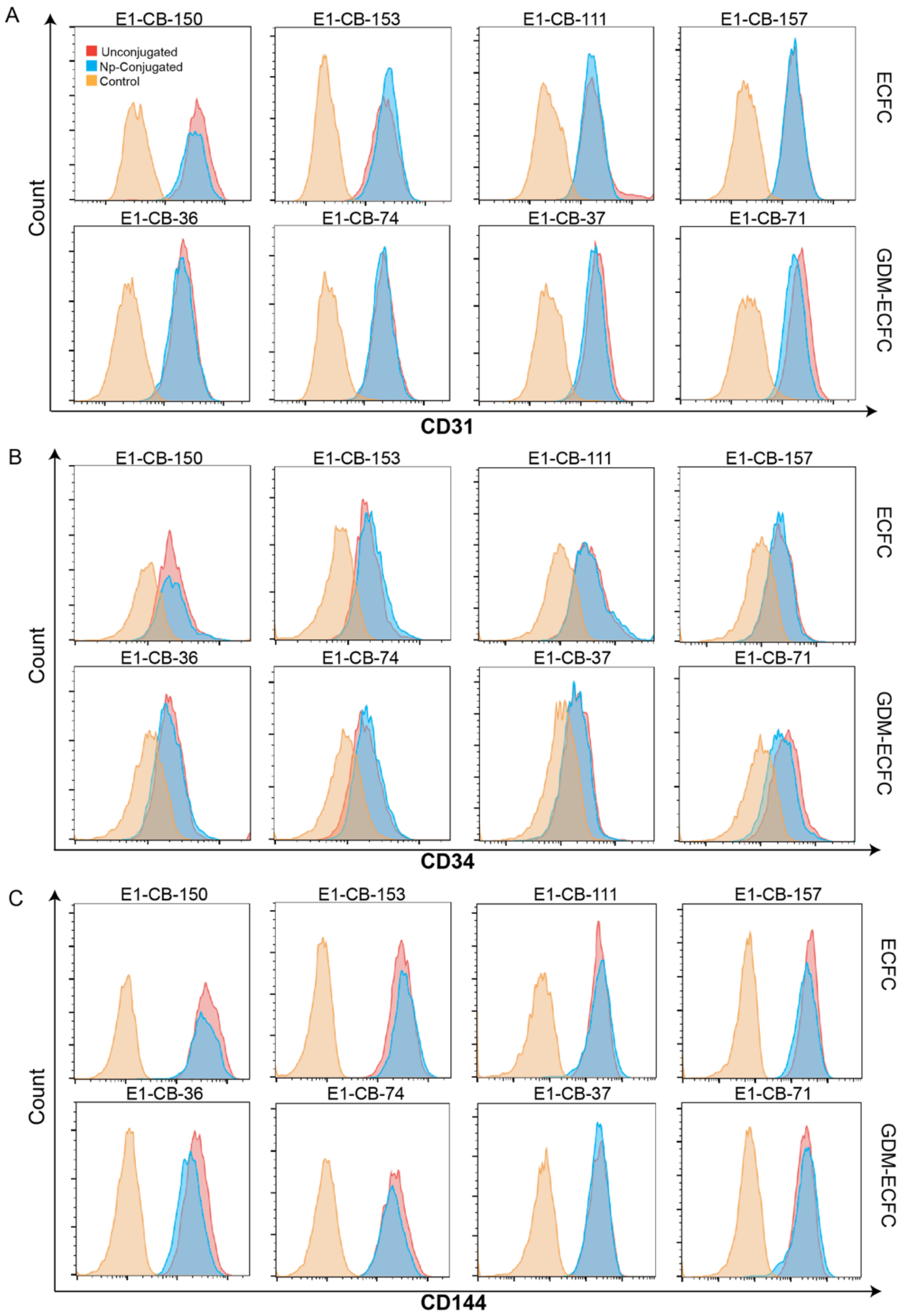

Supplementary Figure 6. Representative histograms for CD31, CD34, and CD144 expression for normal ECFCs (n=4 biological replicates) and GDM-ECFCs (n=4 biological replicates). Blue histograms indicate the NP-conjugated cells, red histograms indicate the unconjugated cells, and yellow histograms indicate the isotype controls.

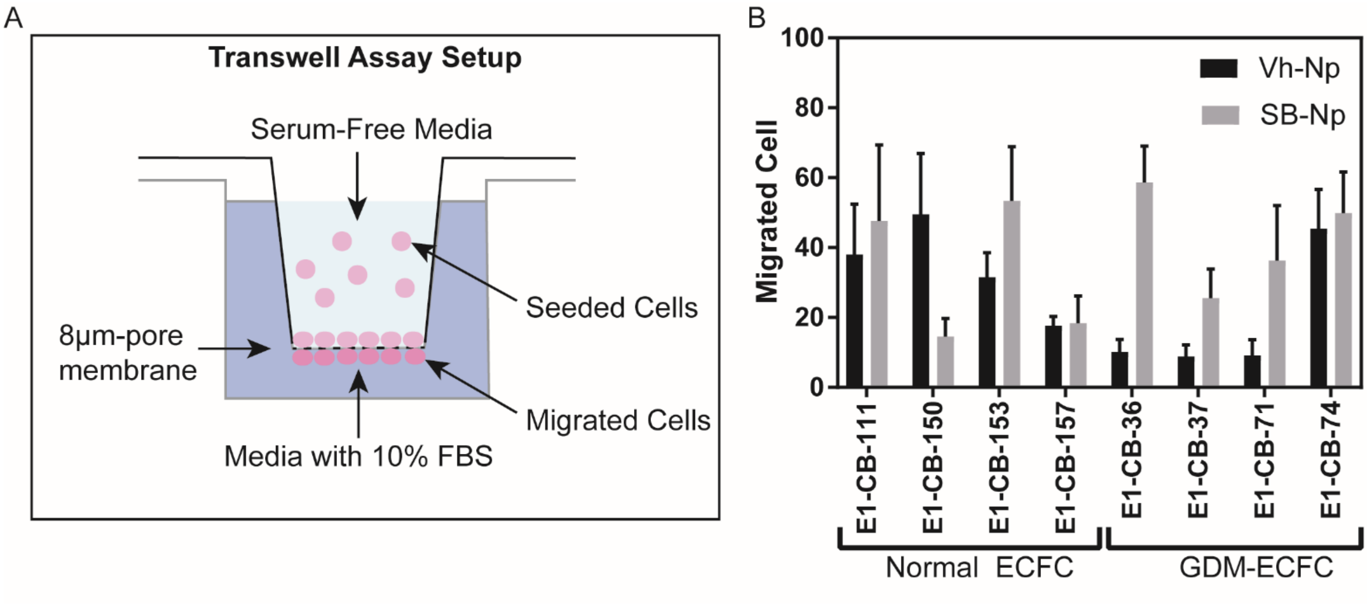

Supplementary Figure 7. (**A**) Transwell assay setup for studying cell migration (**B**) Graph compared migrations of all cell lines treated with Vh-NPs and SB-NPs.

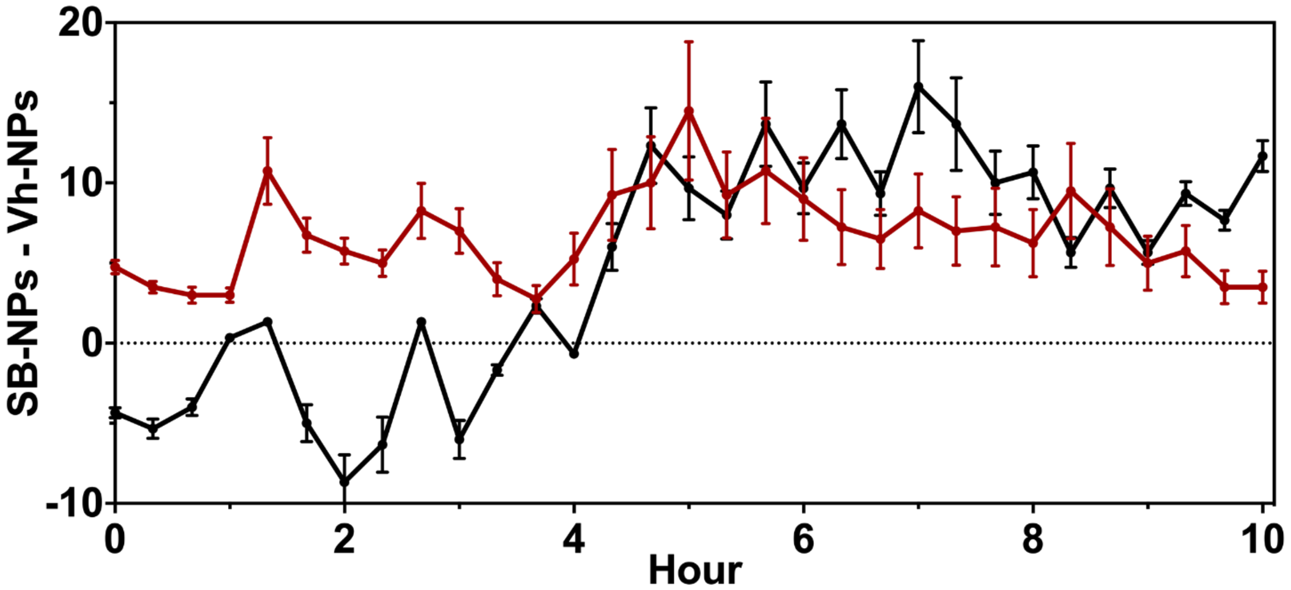

Supplementary Figure 8. Differences between the mean closed networks of the two experimental groups (SB-NPs – Vh-NPs) for normal ECFCs (black line) and GDM-ECFCs (red line) with the pointwise 95 confidence interval (CI) represented by the error bars. A significant difference between the curves for a specific time point is detected if the CI of the difference curve does not cross the reference line (y=0) at that time point.

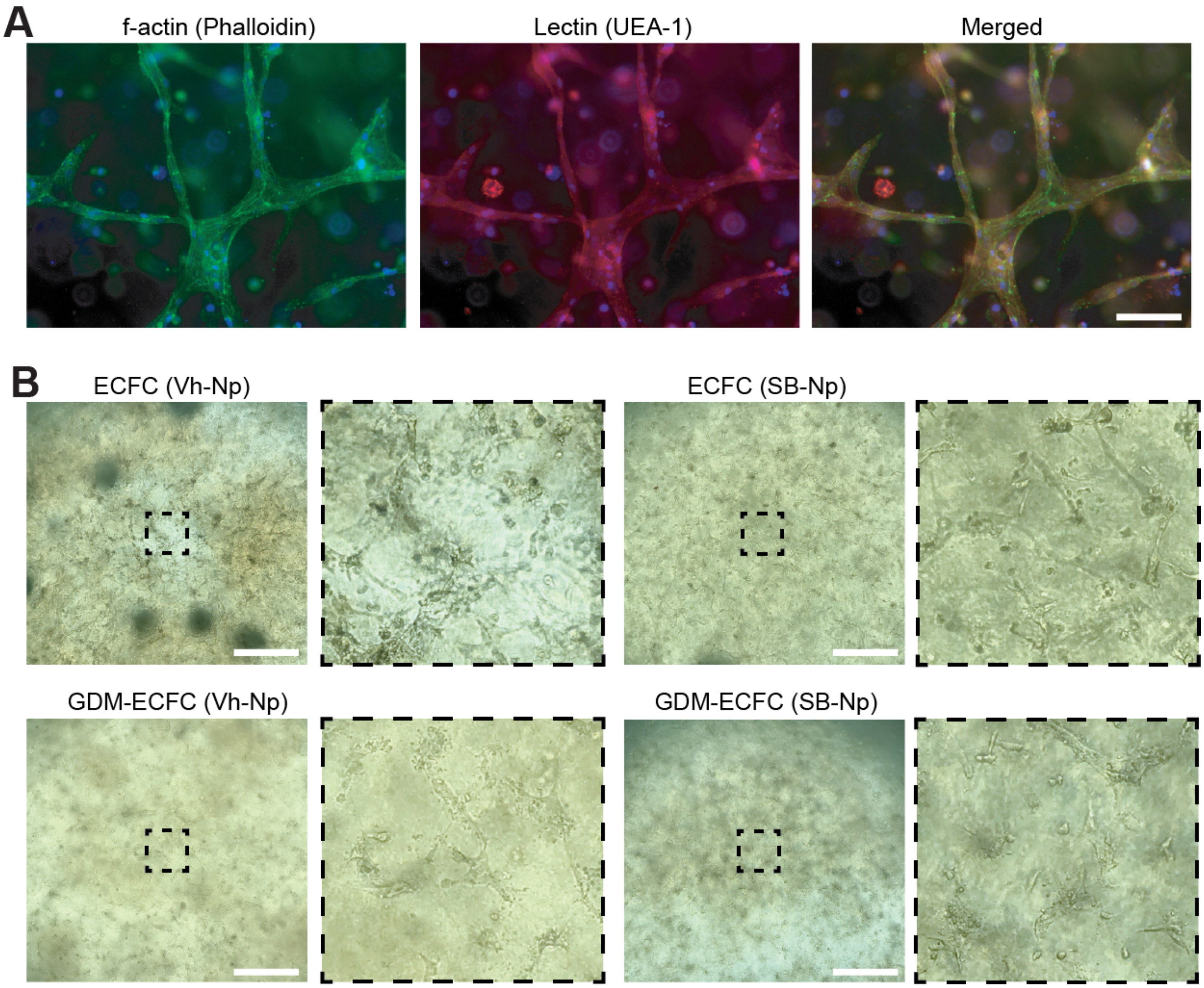

Supplementary Figure 9. Vasculogenic potential of ECFCs in collagen/fibronectin gels. (**A**) Fluorescent photographs of ECFCs forming branching network and lumen within collagen/fibronectin gel after 24 hr culture. Cells were stained for F-actin (Phalloidin, green) and Rhodamine-conjugated *Ulex Europaeus Agglutinin I* (UEA-1, red). Scale bar is 100µm. (**B**) Brightfield images of the network forming within collagen/fibronectin gel from ECFC treated with either Vh-Np or SB-Np after 24 hr culture (4X magnification). Scale bars are 50µm

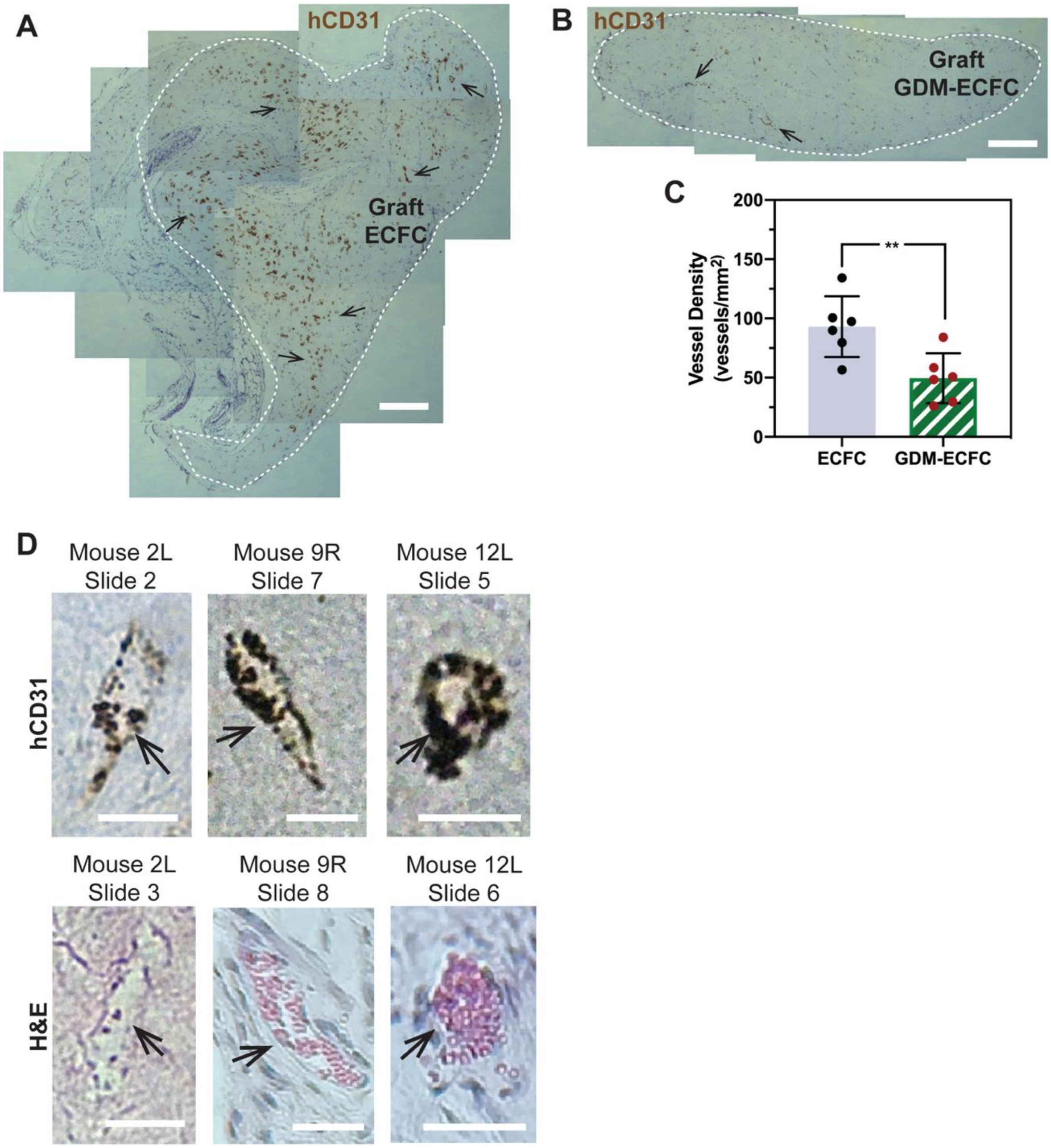

Supplementary Figure 10. Vasculogenic potential of normal and GDM ECFCs *in vivo*. The cell-containing collagen/fibronectin gel plug was implanted subcutaneously within NOD/SCID mice and harvested after 14 days. Representative human CD31 stained grafts containing (**A**) normal ECFCs and (**B**) GDM-ECFCs. Scale bars are 500 µm. (**C**) Quantification of the vessel density demonstrates a decrease in vessel density for grafts containing GDM-ECFCs compared to normal-ECFCs control (**P=0.0093). (**D**) Representative images of vessels with various shapes and sizes, which were analyzed using consecutive slides that were stained with anti-human CD31 (brown, *top*) and H&E (blue and pink, *bottom*). Black arrows indicate human $CD31^+$ vessels that were perfused with mouse RBC. Functional vessels were counted only if they contained at least 1 mouse RBC. Scale bars are 50 µm.

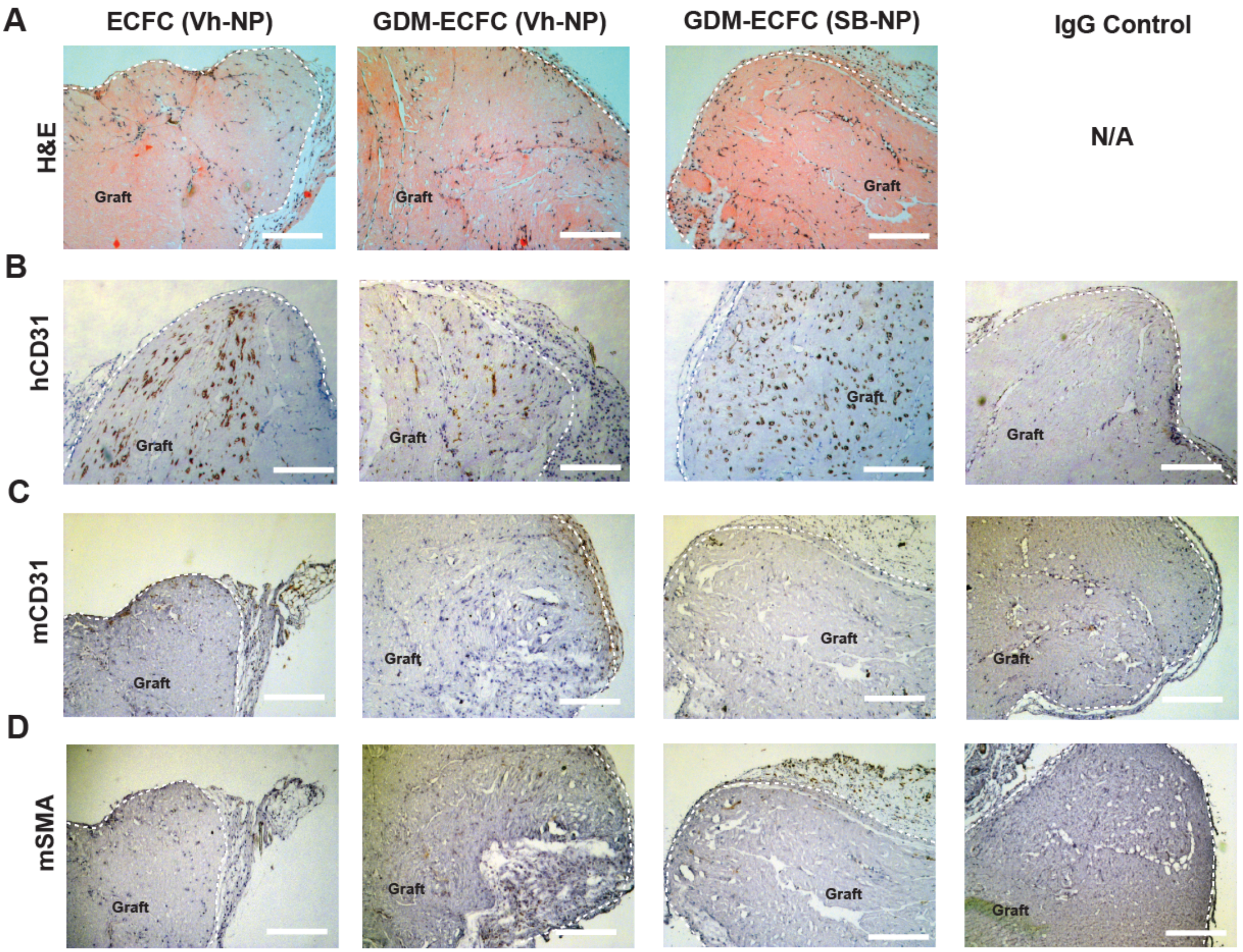

Supplementary Figure 11. Histological and Immunohistochemistry analysis. The cell-containing collagen/fibronectin gel plug was implanted subcutaneously within NOD/SCID mice and harvested after 14 days. Grafts containing ECFC (Vh-NP). GDM-ECFC (Vh-NP), or GDM-ECFC (SB-NPs) were stained for (**A**) H&E, (**B**) human CD31, (**C**) mouse CD31, and (**D**) mouse smooth muscle actin (SMA), as well as their corresponding IgG isotype controls. Scale bars are 100 µm.

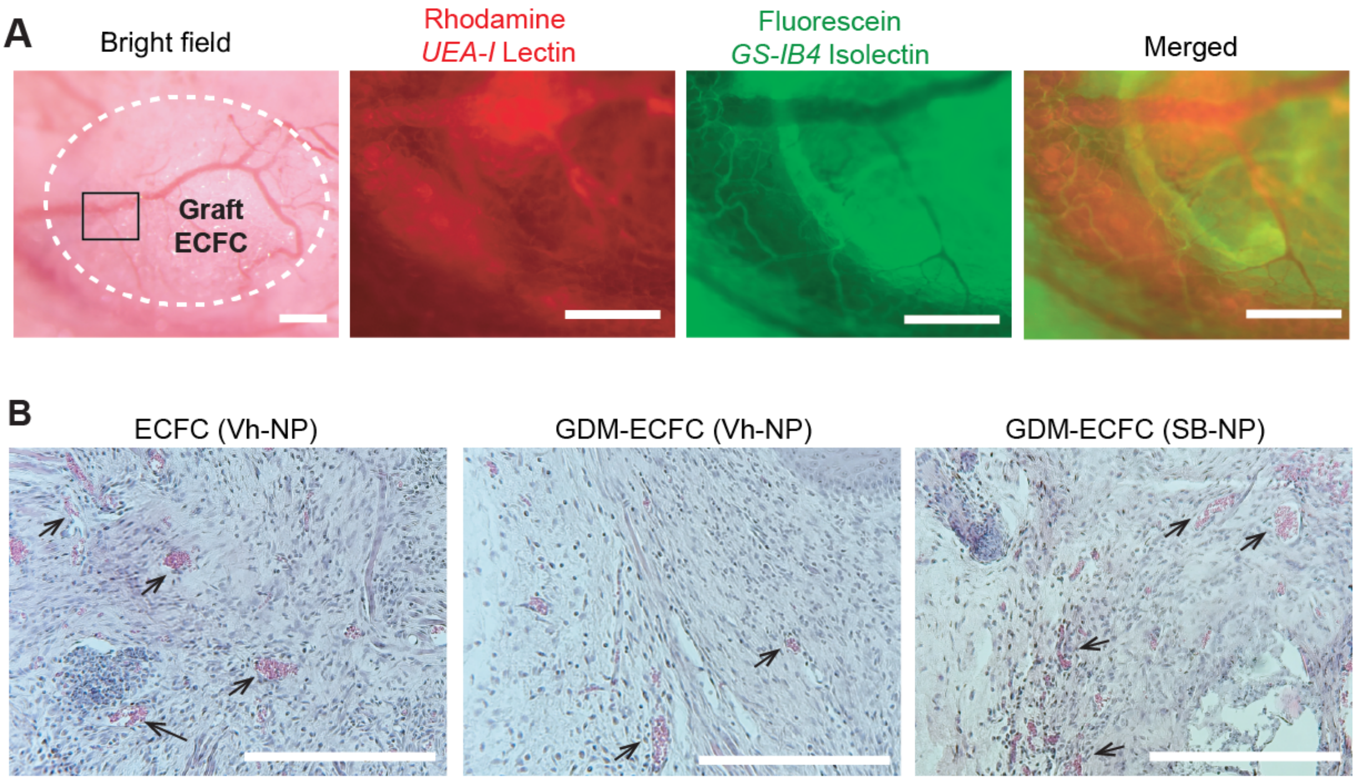

Supplementary Figure 12. Perfusion study and intravital image analysis. (**A**) Intravital images were taken following perfusion study with rhodamine-conjugated UEA-I lectin (human) and fluorescein-conjugated GS-IB4 isolectin (mouse). The bright field image show the graft area (dotted white line). Fluorescent images were taken at 12X magnification from the indicated black square box. Scale bars are 50 µm. At the end of the experiments, the grafts containing (**B**) ECFC (Vh-NP), (**C**) GDM-ECFC (Vh-NP), or (**D**) GDM-ECFC (SB-NP) were fixed and processed for H&E analysis. Representative images demonstrated chimeric vessels containing with murine erythrocytes. Scale bars are 200 µm.

Supplementary Table 1. Clinical data on the cord blood ECFCs used in these studies.

Donor	Condition	Gestational age at birth (wk)	Baby's gender	1 hour GTT
E1-CB-111	Normal	38	Male	102
E1-CB-150	Normal	39	Male	115
E1-CB-153	Normal	39	Male	115
E1-CB-157	Normal	39	Female	139
E1-CB-36	GDM	39	Male	171
E1-CB-37	GDM	40	Male	163
E1-CB-71	GDM	39	Male	153
E1-CB-74	GDM	39	Male	239

Supplementary Table 2. CT values from qRT-PCR and optical density values from western blot assays for quantification of TAGLN expression for each cell line.

Cell lines	CT (Control)	CT (SB 5 μM)	Optical density (Vh-NP)	Optical density (SB-NP)
E1-CB-111	28.12 (±0.14)	30.26 (±0.11)	0.10 (±0.04)	0.20 (±0.13)
E1-CB-150	27.38 (±0.25)	28.71 (±0.11)	0.07 (±0.04)	0.14 (±0.15)
E1-CB-153	26.83 (±0.19)	27.86 (±0.11)	0.24 (±0.34)	0.10 (±0.10)
E1-CB-157	26.00 (±0.22)	28.02 (±0.10)	1.57 (±1.20)	1.24 (±0.79)
E1-CB-36	25.39 (±0.52)	28.65 (±0.19)	1.61 (±0.67)	1.14 (±0.72)
E1-CB-37	24.97 (±0.13)	28.43 (±0.12)	1.44 (±0.87)	1.34 (±1.35)
E1-CB-71	23.75 (±0.42)	26.16 (±0.70)	1.95 (±1.99)	1.55 (±1.30)
E1-CB-74	24.38 (±0.33)	26.43 (±0.18)	1.82 (±0.17)	1.24 (±0.12)

Supplementary Table 3. Flow, Western blot, Immunohistochemical and Immunofluorescence Staining Antibodies used in the studies.

Reagent	Vendor	Host species	Cat. number	Clone number	Dilution factor
CD31 FITC conjugated (Flow)	Abcam	Mouse	ab27333	B-B38	1:100
CD34 PE conjugated (Flow)	Abcam	Mouse	ab187284	ICO-115	1:100
CD144 Alexa Fluor® 488 conjugated (Flow)	Abcam	Rabbit	ab195202	EPR1792Y	1:500
FITC Mouse IgG1 (Flow)	Abcam	Mouse	ab91356	B11/6	1:100
PE Mouse IgG1 (Flow)	Abcam	Mouse	ab91357	B11/6	1:100
AF488 Rabbit IgG (Flow)	Abcam	Rabbit	ab199091	EPR25A	1:500
Transgelin (WB)	Abcam	Rabbit	ab14106	N/A	1:10,000
Vinculin (WB)	Sigma	Mouse	SAB4200729	VIN-11-5	1:10,000
CFSE (Flow)	Abcam	N/A	ab113853	N/A	4ug/ml
Oregon Green™ 488 Maleimide (Flow)	ThermoFisher Scientific	N/A	O6034	N/A	1uM
Anti-human CD31 (IHC)	Dako	Mouse	IR610	JC70A	1:50
Anti-human SMA (IHC)	Dako	Mouse	IR611	1A4	1:50
Anti-mouse PECAM1 (IHC)	Santa Cruz	Goat	SC1506	N/A	1:100
Mouse IgG (IHC)	Alpha Diagnostics	Mouse IgG Control	20008	N/A	1:50
Goat IgG (IHC)	Alpha Diagnostics	Goat IgG Control	20011	N/A	1:100
Rabbit IgG (IHC)	Alpha Diagnostics	Rabbit IgG Control	20009	N/A	1:100
UEA-1 Rhodamine conjugated (IF)	Vector Laboratory	N/A	RL-1062	N/A	1:100
GS-IB4 FITC conjugated (IF)	Vector Laboratory	N/A	FL-1201	N/A	1:100
Anti-mouse SMA (IHC)	Santa Cruz	Rabbit	SC53015	CGA7	1:100

Supplementary Movie 1. Wound healing assay for ECFCs conjugated with Vh-NP.

Supplementary Movie 2. Wound healing assay for ECFCs conjugated with SB-NP.

Supplementary Movie 3. Wound healing assay for GDM-ECFCs conjugated with Vh-NP.

Supplementary Movie 4. Wound healing assay for GDM-ECFCs conjugated with SB-NP.

Supplementary Movie 5. Tube formation assay for ECFCs conjugated with Vh-NP.

Supplementary Movie 6. Tube formation assay for ECFCs conjugated with SB-NP.

Supplementary Movie 7. Tube formation assay for GDM-ECFCs conjugated with Vh-NP.

Supplementary Movie 8. Tube formation assay for GDM-ECFCs conjugated with SB-NP.
