# Supplementary material for: Engineering bioactive nanoparticles to rejuvenate vascular progenitor cells
Source: Commun Biol. 2022 Jun 29;5:635. doi: 10.1038/s42003-022-03578-4 (PMC9243106; doi:10.1038/s42003-022-03578-4)
Supplement: Supplementary file 3 — Description of Additional Supplementary Files [file 42003_2022_3578_MOESM3_ESM.pdf]

Description of Additional Supplementary Files

File name: Supplementary Data 1
Description: The source data behind the graphs in the paper.

File name: Supplementary Movie 1
Description: Wound healing assay for ECFCs conjugated with Vh-NP. The source data behind Figure 4c-e

File name: Supplementary Movie 2
Description: Wound healing assay for ECFCs conjugated with SB-NP. The source data behind Figure 4c-e

File name: Supplementary Movie 3
Description: Wound healing assay for GDMECFCs conjugated with Vh-NP. The source data behind Figure 4c-e

File name: Supplementary Movie 4
Description: Wound healing assay for GDMECFCs conjugated with SB-NP.The source data behind Figure 4c-e

File name: Supplementary Movie 5
Description: Tube formation assay for ECFCs conjugated with Vh-NP. The source data behind Figure 5a-c

File name: Supplementary Movie 6
Description: Tube formation assay for ECFCs conjugated with SB-NP.The source data behind Figure 5a-c

File name: Supplementary Movie 7
Description: Tube formation assay for GDMECFCs conjugated with Vh-NP. The source data behind Figure 5a-c

File name: Supplementary Movie 8
Description: Tube formation assay for GDMECFCs conjugated with SB-NP. The source data behind Figure 5a-c
