# Supplementary material for: Engineering bioactive nanoparticles to rejuvenate vascular progenitor cells
Source: Commun Biol. 2022 Jun 29;5:635. doi: 10.1038/s42003-022-03578-4 (PMC9243106; doi:10.1038/s42003-022-03578-4)
Supplement: Supplementary file 13 — Reporting summary [file 42003_2022_3578_MOESM13_ESM.pdf]

nature portfolio

Corresponding author(s): Donny Hanjaya-Putra

Last updated by author(s): 05/09/2022

Reporting Summary

Nature Portfolio wishes to improve the reproducibility of the work that we publish. This form provides structure for consistency and transparency in reporting. For further information on Nature Portfolio policies, see our Editorial Policies and the Editorial Policy Checklist.

Statistics

For all statistical analyses, confirm that the following items are present in the figure legend, table legend, main text, or Methods section.

n/a	Confirmed	
☐	☒	The exact sample size (*n*) for each experimental group/condition, given as a discrete number and unit of measurement
☐	☒	A statement on whether measurements were taken from distinct samples or whether the same sample was measured repeatedly
☐	☒	The statistical test(s) used AND whether they are one- or two-sided<br>*Only common tests should be described solely by name; describe more complex techniques in the Methods section.*
☒	☐	A description of all covariates tested
☒	☐	A description of any assumptions or corrections, such as tests of normality and adjustment for multiple comparisons
☐	☒	A full description of the statistical parameters including central tendency (e.g. means) or other basic estimates (e.g. regression coefficient) AND variation (e.g. standard deviation) or associated estimates of uncertainty (e.g. confidence intervals)
☐	☒	For null hypothesis testing, the test statistic (e.g. *F*, *t*, *r*) with confidence intervals, effect sizes, degrees of freedom and *P* value noted<br>*Give P values as exact values whenever suitable.*
☒	☐	For Bayesian analysis, information on the choice of priors and Markov chain Monte Carlo settings
☒	☐	For hierarchical and complex designs, identification of the appropriate level for tests and full reporting of outcomes
☒	☐	Estimates of effect sizes (e.g. Cohen's *d*, Pearson's *r*), indicating how they were calculated

Our web collection on statistics for biologists contains articles on many of the points above.

Software and code

Policy information about availability of computer code

Data collection	For most studies, no specialized software was used for data collection. Microscopy data was collected with Lionheart FX and analyzed with Gen5 Software (BioTek). qRT-PCR data was collected with QuantStudioTM 5 System (Applied Biosystems). Western Blot data was collected with Bio-Rad image systems and software.
Data analysis	Vasculogenesis was quantified using the KAV plugin in the FIJI program, Flow cytometry data was analyzed using the FlowJo software, All statistical analysis was conducted in GraphPad Prism.

For manuscripts utilizing custom algorithms or software that are central to the research but not yet described in published literature, software must be made available to editors and reviewers. We strongly encourage code deposition in a community repository (e.g. GitHub). See the Nature Portfolio guidelines for submitting code & software for further information.

Data

Policy information about availability of data

All manuscripts must include a data availability statement. This statement should provide the following information, where applicable:

- Accession codes, unique identifiers, or web links for publicly available datasets
- A description of any restrictions on data availability
- For clinical datasets or third party data, please ensure that the statement adheres to our policy

All data generated or analyzed during this study are included in this published article (and its supplementary information files). Additional data and reagents are available from the corresponding author on reasonable request. Relevant data can be accessed using the following link: https://doi.org/10.5061/dryad.zcrjdfnfv.

Field-specific reporting

Please select the one below that is the best fit for your research. If you are not sure, read the appropriate sections before making your selection.

☒ Life sciences ☐ Behavioural & social sciences ☐ Ecological, evolutionary & environmental sciences

For a reference copy of the document with all sections, see nature.com/documents/nr-reporting-summary-flat.pdf

Life sciences study design

All studies must disclose on these points even when the disclosure is negative.

Sample size	Cord blood samples were collected based on the available donor / biological samples (n=4 normal pregnancies and n=4 GDM pregnancies). Samples size was determined based on a Power Analysis to enable the detection of 50% differences in target endpoints. For the in vivo studies, the number of n=4-6 animals/group/endpoint was finally planed.
Data exclusions	No data was excluded from the study. For the ECFCs isolation from cord blood samples, exclusion criteria include T1DM or T2DM, illness known to affect glucose metabolism (i.e., Cushing syndrome, polycystic ovarian syndrome), use of medications that affect glucose metabolism (i.e., dexamethasone), multiple gestation, history of pre-eclampsia, cardiovascular disease, and women carrying fetuses with chromosomal abnormalities.
Replication	All experiments were reproduced to reliably support conclusions stated in the manuscript. All attempts were made to replicate the reproducibility of the studies using the available biological samples. Each assay was conducted with at least three independent experiment conducted in triplicate for each biological sample.
Randomization	All biological samples were collected with inform consent from patients, who were voluntarily recruited to the studies without any bias toward gender, sex, and races. Animals were randomly divided into experimental groups.
Blinding	The investigators were blinded toward data allocation during data collection and analysis.

Reporting for specific materials, systems and methods

We require information from authors about some types of materials, experimental systems and methods used in many studies. Here, indicate whether each material, system or method listed is relevant to your study. If you are not sure if a list item applies to your research, read the appropriate section before selecting a response.

Materials & experimental systems

n/a	Involved in the study	
☐	☒	Antibodies
☐	☒	Eukaryotic cell lines
☒	☐	Palaeontology and archaeology
☐	☒	Animals and other organisms
☐	☒	Human research participants
☒	☐	Clinical data
☒	☐	Dual use research of concern

Methods

n/a	Involved in the study	
☒	☐	ChIP-seq
☐	☒	Flow cytometry
☒	☐	MRI-based neuroimaging

Antibodies

Antibodies used	All antibodies information is available in the Supplementary Information.
Validation	Each antibody has been benchmarked and validated using the appropriate isotype controls, as well as negative and positive controls. They have been used in our labs to routinely characterize the surface expression of vascular markers in ECFCs.

Eukaryotic cell lines

Policy information about cell lines

Cell line source(s)	ECFCs were isolated from cord blood samples collected from normal and GDM patients following an approved IRB protocol.
Authentication	Each ECFC line was validated in Angio BioCore using previously published in vitro and in vivo vasculogenic assays.
Mycoplasma contamination	All cell lines involved in this study have been screened negative for mycoplasma contamination during periodic testing using Lonza Mycolert Mycoplasma Detection Kit.

Commonly misidentified lines (See ICLAC register)	N/A

Animals and other organisms

Policy information about studies involving animals; ARRIVE guidelines recommended for reporting animal research

Laboratory animals	The study used 8-weeks-old NOD/SCID mice (male and female).
Wild animals	Study did not involve wild animals.
Field-collected samples	Study did not involve field-collected samples
Ethics oversight	The study has been approved by IACUC protocols from the University of Notre Dame and IUSM.

Note that full information on the approval of the study protocol must also be provided in the manuscript.

Human research participants

Policy information about studies involving human research participants

Population characteristics	Human cord blood samples were collected at the time of birth for normal and GDM pregnancies (gestational age 38-42 weeks) following written informed consent. GDM was defined per American College of Obstetrics and Gynecology guidelines. Exclusion criteria include T1DM or T2DM, illness known to affect glucose metabolism (i.e., Cushing syndrome, polycystic ovarian syndrome), use of medications that affect glucose metabolism (i.e., dexamethasone), multiple gestation, history of pre-eclampsia, cardiovascular disease, and women carrying fetuses with chromosomal abnormalities.
Recruitment	Participants were voluntarily recruited to the study. Historical and clinical data were obtained at each visit and at the time of delivery. The Institutional Review Board at the Indiana University School of Medicine (IUSM) approved all protocols, and informed consent was obtained from all women.
Ethics oversight	The study has been approved by IRB protocol from IUSM.

Note that full information on the approval of the study protocol must also be provided in the manuscript.

Flow Cytometry

Plots

Confirm that:

- [x] The axis labels state the marker and fluorochrome used (e.g. CD4-FITC).
- [x] The axis scales are clearly visible. Include numbers along axes only for bottom left plot of group (a 'group' is an analysis of identical markers).
- [x] All plots are contour plots with outliers or pseudocolor plots.
- [x] A numerical value for number of cells or percentage (with statistics) is provided.

Methodology

Sample preparation	ECFCs were collected and cultured from cord blood. Suspended cells were washed twice with 1% BSA in PBS, then stained with the antibodies (1µg/ml) for 30 min at room temperature. The cells were again washed twice with 1% BSA in PBS. The corresponding isotype controls were used for each antibody as described in the Supplementary Information.
Instrument	BD LSR Fortessa X-20 was used to collect the data.
Software	FlowJo software was used to analyzed the FACS data.
Cell population abundance	Cell sorting not employed.
Gating strategy	Using the FCS/SSC gating, debris was removed by gating on the main cell population. Positivity threshold for each cell line was defined on the basis of mock-treated (DMSO) sample compare to the Isotype control. Identical positivity threshold was applied to all samples within cell line.

- [x] Tick this box to confirm that a figure exemplifying the gating strategy is provided in the Supplementary Information.
